# Editorial: Frontiers in the midlands society of physiological sciences (2023-2024)

**DOI:** 10.3389/fphys.2025.1654264

**Published:** 2025-07-09

**Authors:** Sathish Kumar Natarajan, Hong Zheng, Surabhi Chandra, Harold D. Schultz, William C. W. Chen

**Affiliations:** ^1^ Department of Nutrition and Health Sciences, University of Nebraska−Lincoln, Lincoln, NE, United States; ^2^ Division of Biomedical and Translational Sciences, Sanford School of Medicine, University of South Dakota, Vermillion, SD, United States; ^3^ Department of Biology, University of Nebraska at Kearney, Kearney, NE, United States; ^4^ Department of Cellular and Integrative Physiology, College of Medicine, University of Nebraska Medical Center, Omaha, NE, United States

**Keywords:** cardiovascular system, heart failure, artificial intelligence, machine learning, neuroscience, metabolism, endocrine, corpus luteum

**FIGURE 1:**
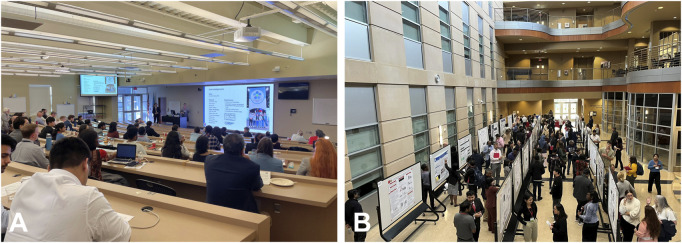
Representative Photos from the 6^th^ MSPS Annual Meeting (2024). This event took place in the Lee Medicine and Science building at the University of South Dakota in Vermillion, South Dakota, United States on 19 October 2024. **(A)** A photo showing one of the talks delivered by a local speaker (photo courtesy of Dr. Jee-Yeon Hwang). **(B)** A photo showing the scientific discussions during the poster session.

This Research Topic covers original studies and reviews submitted by participating members of the Midlands Society of Physiological Sciences (MSPS) from 2023 to 2024. MSPS is a regional nonprofit organization affiliated with the American Physiological Society (APS). MSPS members primarily represent academic and healthcare institutions in Nebraska and South Dakota, advocating for the importance of physiology in education, research, career development, and public awareness. Research within MSPS encompasses a wide range of disciplines and fields, spanning from molecular to whole-organism interrogation in both physiological and disease conditions Figure 1.

Basic and translational cardiovascular sciences have been major research focuses within MSPS for decades. Herein, Lira et al. established a “double-hit” protocol that induces heart failure (HF) with preserved ejection fraction (HFpEF) in FVB/N mice with dysfunction of the myocardial ubiquitin-proteasome system, suggesting its pathogenic role in HFpEF development and/or progression. This protocol simultaneously induces metabolic syndrome and hypertension, producing key features of HFpEF mimicking the clinical syndrome in patients, such as glucose intolerance, elevated blood pressure, and concentric left ventricular hypertrophy with preserved systolic function.

Besides, Zhang et al. interrogated the dose-dependent effects of single interleukin-10 treatment (IL-10) on macrophage phenotype regulation and cardiac remodeling after myocardial infarction (MI). This study demonstrated that an intermediate dose range of IL-10, but not high doses, effectively shifts macrophages toward an anti-inflammatory, reparative phenotype and facilitate cardiac recovery post-MI. Particularly, 250 ng IL-10 exhibited the best overall outcome, whereas ≥1,000 ng IL-10 triggered mixed responses transiently perturbing inflammatory responses and impairing cardiac function, underscoring the importance of IL-10 dosage to maximize its benefits while minimizing potential adverse effects.

Kitzerow et al. used a rat model of MI to map and quantify plasma extravasation (PEx) in multiple organs over time, revealing that PEx occurs in a stage‐ and organ‐specific manner during the progression of chronic HF. Cardiac PEx was highest in the early phase but persisted in scar regions, whereas lung PEx remained significantly elevated,. Abdominal organs such as the liver, pancreas, and spleen showed delayed and increasing PEx correlating with advancing HF. This study suggests that the differential timing and severity of vascular permeability in various organs may contribute to the multi‐organ dysfunction observed in chronic HF.

Further, MSPS members leverage recent advances in artificial intelligence (AI) and machine learning (ML) that are revolutionizing medical practice to accelerate translational and clinical research development. Chowdhury et al. reviewed the clinical progress and challenges of stem cell therapy for HF, noting that while numerous clinical trials within the past decade have shown encouraging results in cardiac repair, significant limitations remain due to discrepancies between preclinical models and human outcomes. The authors highlight that advances in multiomics, precision medicine, and AI/ML can help overcome these hurdles by optimizing cell quality, targeting, and personalized treatment strategies, outlining a roadmap for next-generation stem cell-based therapies that integrate cutting-edge technologies to improve both the structural and functional recovery in HF patients.

In addition, many MSPS members work on basic and translational neurosciences, brain-organ axes, sex differences, and neuropsychiatry. For example, Boomer et al. reported a novel role of the regulators of G-protein signaling 2 (RGS2) in the hypothalamic paraventricular nucleus (PVN) that critically regulates sympathetic activities in a rat model of hypertension. With central adenoviral RGS2 transfection, the authors found that the action of RGS2 on angiotensin-II-induced Gq-protein activation modulated blood pressure, sympathetic activities, and kidney function, providing new insights into the central regulation of blood pressure and potential targets to treat hypertension. Kamra et al. investigated sex-based differences in chemoreflex activation under acute lung injury (ALI), utilizing a bleomycin-induced ALI animal model. The authors highlighted male vs. female chemoreflex changes during the recovery from ALI and emphasized the importance of sex as a determinant in respiratory responses. These findings point to a need for a broader understanding of sex-based variations in lung disorders and underscore the significance of sex as a crucial factor in respiratory research.

Ma et al. described a key role of a genetic polymorphism of brain-derived neurotrophic factor (BDNF) that possibly mediates the sexual difference in idiopathic autism spectrum disorder (ASD) and human autism. Using humanized mice with genetic knock-in of the target human BDNF methionine (Met) allele, the authors showed that diminished activity-dependent BDNF signaling resulted in an increased excitability of prefrontal cortex pyramidal neurons in male, but not female, BDNF^+/Met^ mice. This study revealed the necessity of examining sex as a biological variable in neuropsychiatric disorders and provides avenues for exploring targeted therapies for genetic variations in neural development.

Moreover, MSPS members have worked on metabolic and endocrine physiology for many years. Here, Evans et al. examined the correlation between leptin resistance and cardiac vagal postganglionic (CVP) neuronal dysfunction in type 2 diabetes mellitus (T2DM). Leptin acts on pro-opiomelanocortin (POMC) neurons to regulate satiety. However, in T2DM, leptin resistance develops along with complications such as CVP neuronal dysfunction. In a rodent model using a high-fat diet and low-dose streptozotocin, the authors observed reductions in leptin receptor expression and uncoupling protein 2 in CVP neurons as T2DM progressed and reduced cardiac parasympathetic activity, possibly due to neuronal remodeling and insulin resistance.

Monaco and Davis reviewed the angioregression of corpus luteum and factors associated with luteolysis on luteal vasculature. This review article is an excellent summary of the work in Dr. John S. Davis’s laboratory at the University of Nebraska Medical Center (UNMC) and other research groups focusing on corpus luteum in the last 3 decades. This article extensively summarizes the essential roles of pro-angiogenic, anti-angiogenic, and pro-inflammatory factors involved in luteal angioregression, such as vascular endothelial growth factor, thrombospondin 1, and tumor necrosis factor alpha. Besides, this review outlines the underlying mechanisms in luteal angioregression, including vascular physiology and endothelial cell apoptosis.

Overall, the articles contained in this Research Topic fully reflect the width and depth of the research among MSPS members. MSPS will continue to promote the creation, application, and translation of novel theories, innovative methodologies, and advanced therapeutics to support the development of modern physiology and to improve human health and welfare in America’s Heartland.

